# Bladder preservation approach versus radical cystectomy for high-grade non-muscle-invasive bladder cancer: a meta-analysis of cohort studies

**DOI:** 10.1186/s12957-018-1497-0

**Published:** 2018-10-02

**Authors:** Pei-lin Shen, Ming-en lin, Ying-kai Hong, Xue-jun He

**Affiliations:** 1 Department of Urology, The First Affiliated Hospital of Shantou University Medical College, No. 57, Changping Road, Jinping District, Shantou, Guangdong China; 2 Shantou University Medical College, No. 22, Xinling Road, Jinping District, Shantou, Guangdong China

**Keywords:** Bladder preservation, High-grade non-muscle-invasive bladder cancer, Radical cystectomy, Meta-analysis

## Abstract

**Background:**

High-grade non-muscle-invasive bladder cancer is superficial; nonetheless, it is an aggressive cancer. Proper management strategy selection following transurethral resection between bladder preservation (BP) and radical cystectomy (RC) could result in delayed or excessive treatment. Hence, selecting the optimal treatment modality remains controversial to date.

**Methods:**

We searched MEDLINE, The Cochrane Library, EMBASE, China National Knowledge Infrastructure, and Wanfang database through 12 April 2018. Quality and publication bias were assessed using the Newcastle-Ottawa Scale and Begg’s/Egger’s test. We collected 2-year, 5-year, 10-year, and 15-year survival rate and hazard ratio (HR) for overall survival (OS), cancer-specific survival (CSS), and progression-free survival (PFS). Using the Review Manager 5.2 software, we used the odds ratio (OR) of specific years and HR for meta-analysis. Subgroup analysis was performed by the original tumor state, radical cystectomy timing, bladder preservation modality, and age.

**Results:**

In total, 11 cohorts with 1735 patients were selected for the meta-analysis. All OR of OS supported BP as a better treatment option; however, all OR of PFS had no significant differences. As for CSS, only the 15-year OR reflected a statistical significance preferring RC. Subgroup analysis showed that BP is more appropriate for patients older than 65 and G3 tumor. Limited data demonstrated that late RC (> 3 months) is more effective compared to early RC (< 3 months) and intravesical Bacillus Calmette–Guerin was not statistically different from that of RC. The mixed BP modalities were significantly better compared to RC in OS and worse in CSS, with both having a very low evidence strength.

**Conclusions:**

BP is a superior treatment modality compare to RC, especially for older patients and T1G3 or lower grade tumors. However, the superior BP modality was unclear. Conversely, RC could be a better option for younger patients. More specifically, late RC may be more beneficial but had a very-low-level of evidence. Quality of life should be considered equal to survival outcome; hence, post-treatment follow-up needs to be performed. Prospective randomized studies should be performed to overcome the limitations of this meta-analysis study.

**Registration:**

Registration ID is CRD42018093491.

## Background

Bladder cancer (BC) has a high morbidity in patients worldwide. As the 9th most commonly diagnosed cancer and the 13th most common cause of death worldwide, BC caused 188,000 deaths in 2015 worldwide [1, 2].

Among the 81,190 estimated newly diagnosed BC in the US in 2018, nearly 75% were non-muscle-invasive bladder cancer (NMIBC). NMIBC is defined as a superficial neoplasia confined to the mucosa, (including Ta which is a noninvasive papillary carcinoma and carcinoma in situ (CIS) which is flat and non-papillary) or lamina propria (T1) based on the American Joint Committee on Cancer (AJCC) staging system, also known as the tumor node metastases (TNM) classification [3–5]. Histologically, BC is generally graded using the 1973 World Health Organization (WHO) classification system or the 2004 revision. The 1973 version comprises grade 1 to 3 and is based on the degree of cellular anaplasia compatible for the diagnosis of malignancy. Grade 1 (G1) applies to tumors having the least degree, grade 3 (G3) applies to tumors having the most severe degree, and grade 2 (G2) lies in between. The 2004 revision categorizes tumors into “low-grade (LG)” or “high-grade (HG)” depending on the neoplasm of the urothelium lining papillary fronds. This may show either an orderly appearance or with easily recognizable variations in architecture and cytologic features or a predominant pattern of disorder with moderate-to-marked architectural and cytologic atypia [6, 7]. Both grading systems have confirmed prognostic value and have been accepted widely. However, which one is more clinically significant remains controversial [8–10]. In NMIBC, high-grade non-muscle-invasive bladder cancer (HGNMIBC) has the highest risk due to its aggressive clinical, biologic, and histopathologic characteristics [11, 12]. A 15-year study demonstrated that high-grade T1 bladder cancer had a 50% progression rate and had a mortality of 30% during patient follow-up [13]. Hence, the management strategy for HGNMIBC is an unmet clinical need.

Regarding bladder preservation (BP), intravesical Bacillus Calmette–Guerin (BCG) following transurethral resection (TUR) has been the gold standard for over 40 years and has been demonstrated to decrease recurrence rates, progression rates, and mortality in high-risk NMIBC patients [14–16]. However, several studies have reported that 23 to 74% high-grade T1 tumor recurred and more than 50% progressed after receiving intravesical BCG therapy [17–20]. The side-effects of BCG are common and can be severe; hence, new bladder preservation approaches for HGNMIBC should aim to provide better quality of life (QoL). However, clinical data on the different kinds of intravesical instillation, chemotherapy, radiotherapy, device assisted therapy, and electromotive drug administration is limited [21]. In addition, BP may cause inadequate treatment due to the delay of radical cystectomy (RC). About one third of patients treated with intravesical BCG still undergo RC during the treatment procedure [22]. Delay in RC may increase the risk of lymph node metastases and even bladder cancer-specific mortality [23, 24]. Thus, timely RC has to be performed during HGNMIBC treatment.

As HGNMIBC has a risk of recurrence and progression, RC has become a popular therapeutic strategy [25, 26]. There were multiple studies demonstrating that RC is the preferred treatment option due to better survival rates [27–29]. However, there is a consensus that RC and intravesical BCG shares the same curative effect and several studies have demonstrated that RC is therapeutically excessive [30]. The RC procedure includes surgically removing the whole bladder along with adjacent organs and reconstructing the urinary drainage, which could cause severe complications and even death. Several studies have reported that many patients suffered from different short-term complications which require more than 6 months for the QoL to normalize to preoperative levels [31]. Because survival and QoL outcomes remain uncertain, the impact of RC still needs to be explored further.

Major associations share similar treatment opinions for HGNMIBC. American Urological Association (AUA) guidelines [32] and European Association of Urology (EAU) guidelines [33] strongly recommends 6 weeks of BCG intravesical instillation for high-risk NMIBC and a maintenance schedule of up to 3 years. The National Comprehensive Cancer Network (NCCN) guidelines [34] also regard BP as the first line treatment option for the management of high-grade tumors. Specifically, observation and intravesical instillation (BCG and chemotherapeutics) should be selected for HG cTa patients whereas BCG is the only recommended BP modality for HG cT1 patients. As for RC, EAU, and NCCN, their guidelines recommend that immediate or early (within 3 months of diagnosis) RC should be considered as an option worth discussing with a low grade of evidence. More specifically, AUA guidelines for grade C suggest after a single course of BCG, RC should be offered for high-grade T1 patients who are fit for surgery and a second course of induction should be considered for persistent or recurrent high-grade Ta or CIS patients. However, NMIBC could be a mixture of CIS, Ta, and T1 instead of containing only one single grade of tumor. Although consensuses exists among the three guidelines, questions regarding BP selection, necessity and timing of RC, and the selection of optimal treatment strategies between BP and RC still remain controversial [35, 36].

To help determine the optimal treatment modality, we performed a meta-analysis of BP versus RC for patients with HGNMIBC. The survival outcomes included overall survival (OS), cancer-specific survival (CSS), and progression-free survival (PFS).

## Methods

### Registration information

This meta-analysis is registered on PROSPERO (https://www.crd.york.ac.uk/PROSPERO). The registration ID is CRD42018093491.

### Eligibility criteria

Eligible studies met the following criteria: (1) The subject of the study were patients suffering from HGNMIBC (including “grade 3” under the 1973 WHO classification and “high-grade” under the 2004 WHO classification). All 1973 WHO G3 tumors are assigned to the 2004 WHO high-grade carcinoma category while revising [37]. (2) Studies focusing on or containing the comparison between radical cystectomy and bladder preservation approach (observation, intravesical BCG, and/or any other kind of intravesical instillation) following TUR. A delayed radical cystectomy was performed if bladder preservation approach failed or the side-effects were intolerable. (3) The data of survival outcomes (OS, CSS, or PFS) were available.

Studies were excluded based on the following criteria: (1) Studies including patients with an original state of BCG refractory, resistance, or failure. (2) The key outcomes were not available. (3) Not intervention research such as review, letter, viewpoint, or case reports. (4) The sample size was less than 20 patients in total or 10 patients in BP/RC group.

### Search strategy

**Fig. 1:**
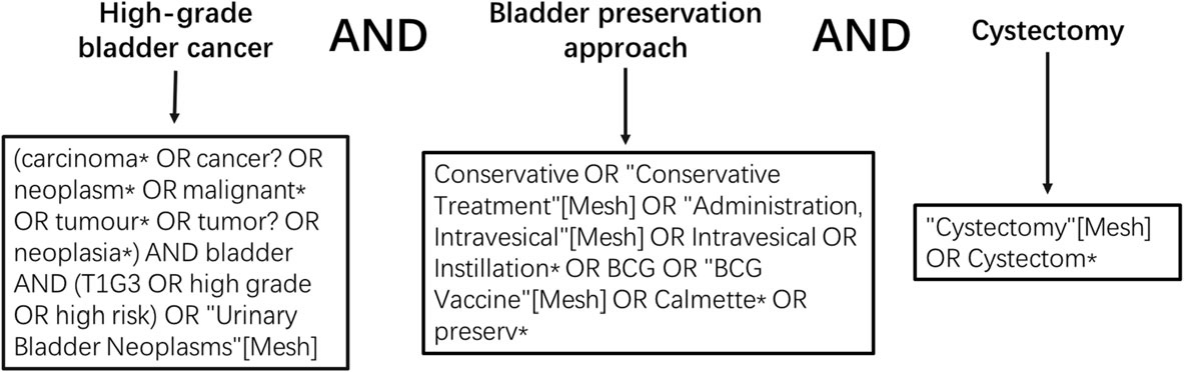
Search strategy of MEDLINE

We performed an internet search for relevant studies from The Cochrane Library, MEDLINE, EMBASE, CNKI (China National Knowledge Infrastructure), and Wanfang database through 12 April 2018. The search strategy was to combine MeSH terms (in MEDLINE and The Cochrane Library) or Emtree terms (in EMBASE) with free words from three English medical databases. As in CNKI and Wanfang database, we searched relative terms as Subject, Keyword, Title, and Abstract, and the explored field was restricted in Medicine and Health. Terms relating to or describing high-grade bladder cancer, cystectomy, and bladder preservation approach (including observation, BCG, intravesical instillation, bladder preservation) were used. There was no filter used for randomized controlled trials or cohort studies and no language restriction was applied. The search strategy for MEDLINE is demonstrated in Fig. 1. The full-text of all published studies including meeting abstracts that met the eligibility criteria were collected. No attempt was made to contact the authors for obtaining or confirming published data.

### Quality assessment

All eligible studies were cohort studies, and the Newcastle-Ottawa Scale (NOS) was used for quality assessment [38]. The scale contained three categories: Selection, Comparability, and Outcome. Each numbered item within the Selection and Outcome were awarded one star. As for the Comparability group, a maximum of two stars were assigned. After the assessment, a total score range from 0 to 9 stars was calculated for every study. Study bias was scaled as high risk of bias (0–3 stars), middle risk of bias (4–6 stars), and low risk of bias (7–9 stars) and represented inferior quality, medium quality, and superior quality respectively.

STATA 12.0 software was used for publication bias assessment. Each comparison was assessed using Egger’s test except for those containing only two studies where the Begg’s test was used. *P* > 0.05 was considered to have no publication bias.

### Data collection process

Data was extracted from each article and included information on country, study interval, original tumor state, radical cystectomy timing, bladder preservation modality, gender composition, study size, age, median follow-up time, and type of outcomes. As for multiple articles by the same author, the latest article was selected for inclusion. Table 2 describes the characteristics of the included studies.

### Synthesis of results

From the selected studies, we collected 2-year, 5-year, 10-year, and 15-year survival rate and hazard ratio (HR) of each outcome. If there was no direct data provided in the publication but a Kaplan–Meier survival curve was present, we extracted information from the curve using the Digitizer 4.1 software and then mathematically calculated the HR and survival rate. Using Review Manager 5.2 software, we used HR and odds ratio (OR) for meta-analysis and subgroup analysis was performed using the original tumor state, radical cystectomy timing, bladder preservation modality, and age. *A p* value less than 0.05 of a pooled HR or OR was considered statistically significant.

Heterogeneity between the studies in effect measures was assessed using the *I*^2^ statistic. An *I*^2^ value greater than 50% was indicative of substantial heterogeneity and hence used the random effect model for synthesis. A fix effect model was used for synthesis if *I*^2^ value was less than 50%.

## Results

### Study selection

**Fig. 2:**
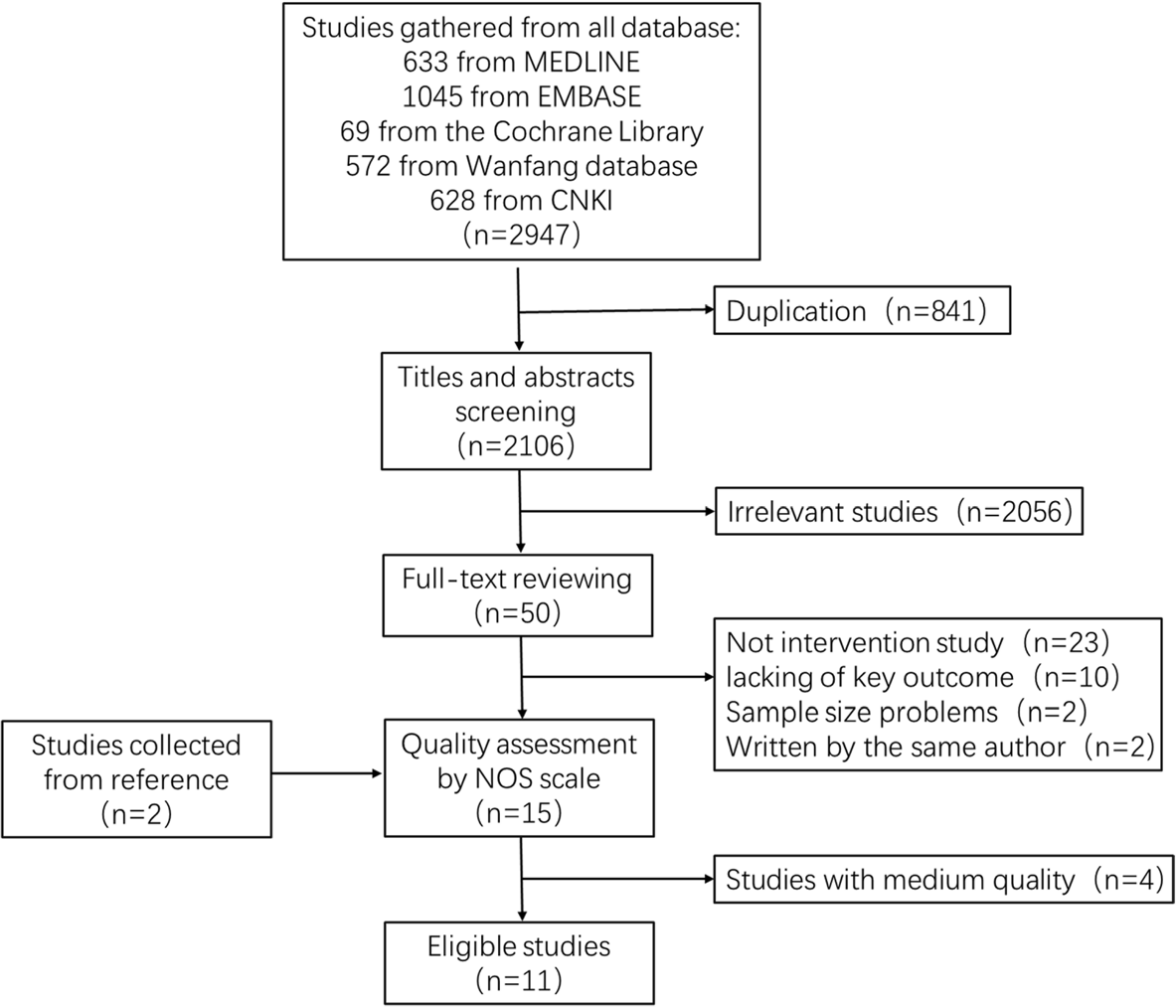
Flow diagram of study selection

A total of 2947 articles (633 from MEDLINE, 1045 from EMBASE, 69 from the Cochrane Library, 572 from Wanfang database, and 628 from CNKI) were assembled from all databases mentioned above. After removing duplicates, 2106 articles remained. By screening the titles and abstracts, 2056 articles were excluded with 50 articles meeting the inclusion criteria or unlikely to be rejected. After a full-text review, 23 articles including reviews, letters, protocols, viewpoints, editorials, comments, and case reports were excluded. Ten studies lacking key outcomes and two with sample size issues (one having 10 patients in total and one having only 7 patients in the RC group) were also excluded. When 2 articles were present from the same author, we chose the latest one for inclusion. Finally, there were 13 studies left, together with 2 studies selected from the references of relative articles. In all, a total of 15 studies were included for further quality assessment. After evaluating each study using the NOS scoring standard, 4 studies were excluded for middle risk of bias. In the end, 11 studies with superior quality were considered eligible for analysis (Table 1). The flow diagram of study selection is shown in Fig. 2.

**Table 1:** Newcastle-Ottawa Scale (NOS) score for included studies

Study	Year	Selection				Comparability		Outcome			Scale
		Exposed	Non-exposed	Ascertainment of exposure	Start without outcome present	Major factor	Addition factor	Outcome assessment	Follow-up length	Adequacy of outcome	
Badalato	2012	⭐	⭐	⭐	⭐	⭐	⭐	⭐	⭐		8
De Berardinis	2011	⭐	⭐	⭐	⭐	⭐	⭐	⭐	⭐		8
Denzinger	2008	⭐	⭐	⭐	⭐	⭐	⭐	⭐	⭐		8
Hautmann	2009	⭐	⭐	⭐	⭐		⭐	⭐			7
Jager	2011	⭐	⭐	⭐	⭐	⭐	⭐	⭐	⭐	⭐	9
Li	2011	⭐	⭐	⭐	⭐	⭐	⭐	⭐	⭐		8
Patard	2001	⭐	⭐	⭐	⭐		⭐	⭐	⭐		7
Spaliviero	2014	⭐	⭐	⭐	⭐	⭐	⭐	⭐			7
Sun	2008	⭐	⭐	⭐	⭐	⭐	⭐	⭐	⭐		8
Thalmann	2004	⭐	⭐	⭐	⭐	⭐	⭐	⭐	⭐		8
Wong	2009	⭐	⭐	⭐	⭐	⭐		⭐	⭐		7

### Study characteristics

From Table 2, the comparison between BP and RC initiated from 2001 worldwide. A total of 1735 patients were selected for analysis. There are 7 studies that used the 1973 WHO grading system [27, 29, 39–43] while 4 used the 2004 revision [28, 44–46]. BP modality mentioned in the included studies were mostly intravesical therapy including BCG, epirubicin, mitomycin, and hydroxycamptothecine, and 2 studies even included observation as a BP strategy [44, 46]. Several studies did not describe the BP modality specifically; however, the majority of the studies selected intravesical BCG as the BP strategy including 7 of them that used BCG only [27, 29, 39–42, 45]. Patients of 6 cohorts received RC within 3 months [27, 29, 39, 41, 44, 46] and 2 other cohorts had surgery with a median time of 4 months [28] and 16 months [42]. Summarizing the eligible data, males had the advantage in gender composition in all studies with a ratio of 1.4–7.2:1. The average patient age in all the cohorts was older than 60. In the majority of the studies, the average age of the BP groups was older compared to the RC groups; however, no statistical significance was detected. The median follow-up time ranged from 38.4 months to 99.6 months. Extracted outcomes were HR and survival rate of specific years including OS from 6 studies, CSS from 10 studies, and PFS from 3 studies.

**Table 2:** Characteristics of included studies

Study year	Country	Study interval	Original tumor state	Radical cystectomy timing	Bladder preservation modality	Male/female (BP vs RC)	Study size (BP vs RC)	Age (BP vs RC)	Median follow-up time (BP vs RC)	Types of outcomes
Hautmann 2009	Germany	1986–1908	T1G3	Within 3 mo	BCG	Ratio 4:1	99 vs 175	63.9	58 mo	CSS PFS
Jager 2011	Germany	1989–2006	HGNMIBC	Median 4 mo	Endoscopic treatment/IVT	236 vs 42	43 vs 235	66	79 mo	CSS
Thalmann2004	Switzerland	1980–1999	T1G3	Within 3 mo	BCG	71/21 vs 26/3	92 vs 29	69 vs 66	82.8 mo	OS CSS PFS
De Berardinis 2011	Italy	1995–2001	T1G3	Within 2 mo	BCG	60/20 vs 49/23	80 vs 72	70.4 vs 69.6	99.6 mo	OS CSS PFS
Denzinger 2008	Germany	1990–2005	T1G3	UN	BCG	95/30 vs 71/27	125 vs 98	73 vs 71	56 mo vs 51 mo	OS CSS
Li 2011	China	1995–2007	T1G3	3 mo	BCG	18/14 vs 10/6	32 vs 16	61 vs 58	66 mo vs 72 mo	OS CSS
Patard 2001	France	1979–1996	T1G3	Median 16 mo	BCG	UN	50 vs 14	62.52 vs 62.78	62 mo	CSS
Sun 2008	China	199–2007	T1G3	UN	BCG/MMC/EPI /HCOT	68/13 vs 23/9	81 vs 32	66 vs 64	64 mo vs 62 mo	OS CSS
Badalato 2012	USA	1990–2010	HGT1	Within 3 mo	Observation /IVT	88/25 vs 168/68	236 vs 113	69.5 vs 68.3	46.4 mo vs 51.5 mo	CSS
Spaliviero 2014	USA	2000–2012	HGT1	Within 3 mo	Observation /BCG	Ratio 3:1	21 vs 15	68	38.4 mo	CSS
Wong 2009	UK	1998–2007	HGNMIBC	UN	BCG	UN	41 vs 36	67.23	53 mo	OS

*Abbreviations*: *BP* bladder preservation, *RP* radical cystectomy, *T1G3* pathological grade 1 under the American Joint Committee on Cancer (*AJCC*) staging system and histological grade 3 under the 1973 World Health Organization (*WHO*) classification, *HGNMIBC* high-grade non-muscle-invasive bladder cancer, *mo* month, *BCG* Bacillus Calmette–Guerin, *EPI* epirubicin, *MMC* mitomycin, *HCPT* hydroxycamptothecine, *IVT* intravesical therapy, *OS* overall survival, *CSS* cancer-specific survival, *PFS* progression-free survival, *UN* unknow

### Comparison of overall survival of bladder preservation versus radical cystectomy

**Fig. 3:**
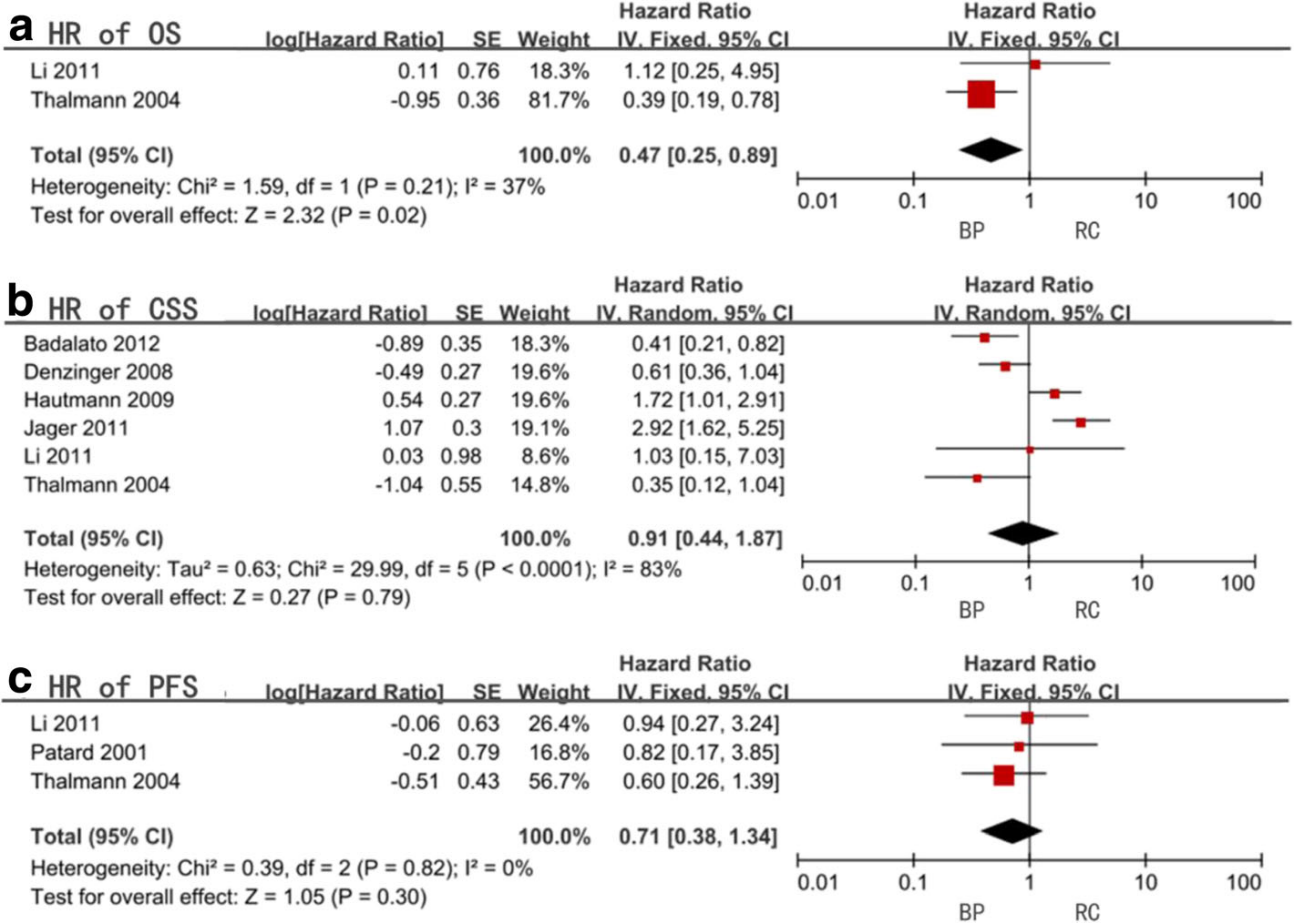
Forest plots of hazard ratio (HR)

Six studies were selected for our OS meta-analysis. Only 2 articles had retrievable HR data and the combined HR, and the 95% confidence interval (CI) was 0.47 (0.25–0.89) with a statistical significance (*P* = 0.02). Heterogeneity was considered acceptable with an *I*^2^ = 37% (Fig. 3a, Table 3). No subgroup analysis was performed due to data unavailability.

**Table 3:** Meta-analysis of hazard ratio (HR)

	Factors		Study size	HR (95%CI, *P* value)	*I*^2^ (%)
OS	All included studies		2	0.47(0.25–0.89,0.02)	37
CSS	All included studies		6	0.91(0.44–1.87,0.79)	83
	Original tumor state	HG	2	1.10(0.16–7.53,0.92)	94
		G3	4	0.99(0.53–1.87,0.98)	59
	Radical cystectomy timing	> 3 months	1	2.92(1.62–5.25,0.0004)	not available
		< 3 months	4	0.71(0.28–1.83,0.48)	78
	Bladder preservation modality	BCG	4	0.81(0.38–1.75,0.59)	72
		Mix	2	1.10(0.16–7.53,0.92)	94
	Age	< 65 years old	2	1.66(0.99–2.76,0.05)	0
		> 65 years old	4	0.74(0.28–1.98,0.55)	88
PFS	All included studies		3	0.71(0.38–1.34,0.3)	0
	Radical cystectomy Timing	> 3 months	1	0.82(0.17–3.85,0.8)	not available
		< 3 months	2	0.69(0.35–1.39,0.3)	0
	Age	< 65 years old	2	0.89(0.34–2.34,0.82)	0
		> 65 years old	1	0.60(0.26–1.39,0.24)	not available

**Fig. 4:**
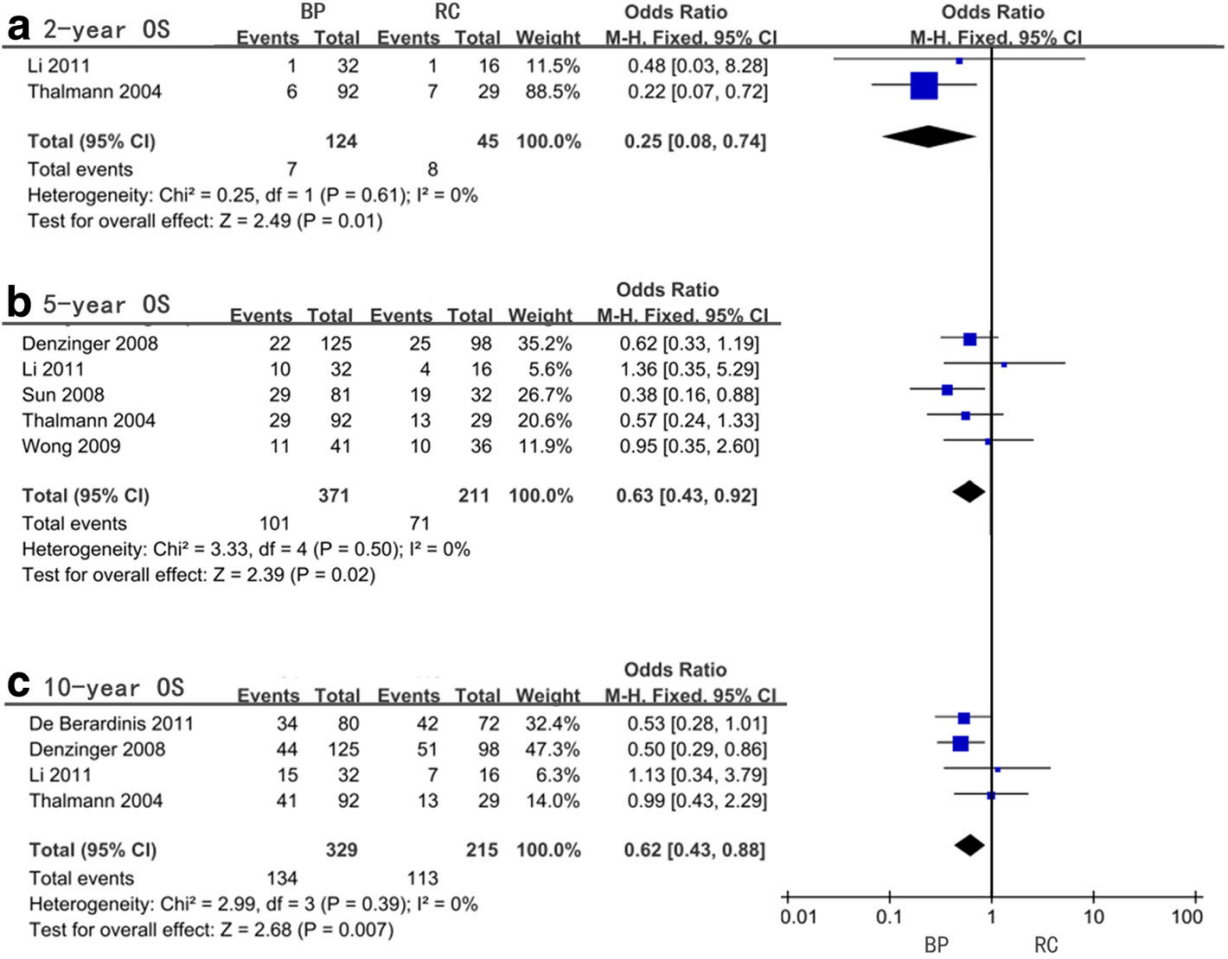
Forest plots of odds ratio (OR) for overall survival (OS)

With regards to the 2-year, 5-year, and 10-year OS, the pooled OR were 0.25 (95%CI = 0.08–0.74, *P* = 0.01, *I*^2^ = 0%), 0.63 (95%CI = 0.43–0.92, *P* = 0.02, *I*^2^ = 0%), and 0.62 (95%CI = 0.43–0.88, *P* = 0.007, *I*^2^ = 0%), respectively (Fig. 4, Table 4). Only one article provided OR of 15-year OS which was 0.35 (95%CI = 0.13–0.95, *P* = 0.04). The subgroup analysis was performed for only the 2-year, 5-year, and 10-year OS because of data deficiency for the 15-year OS and all indicated BP as a better treatment option for patients older than 65 years old (*P* = 0.01, 0.009, and 0.004 respectively). In the 5-year OS, the same result as mentioned before regarding BP was found for the G3 group of subgroup analysis for original tumor state (*P* = 0.01) and mix group of subgroup analysis for bladder preservation modality (*P* = 0.02). Subgroup analysis for all RC timing groups was not carried out. No significant heterogeneity was found for all subgroups (Table 4).

**Table 4:** Meta-analysis of odds ratio (OR) for overall survival (OS)

OS	Factors		Study size	OR (95%CI, *P* value)	*I*^2^ (%)
2-yr	All included studies		2	0.25(0.08–0.74,0.01)	0
	Age	< 65 years old	1	0.48(0.03–8.28,0.62)	not available
		> 65 years old	1	0.22(0.07–0.72,0.01)	not available
5-yr	All included studies		5	0.63(0.43–0.92,0.02)	0
	Original tumor state	HG	1	0.95(0.35–2.60,0.93)	not available
		G3	4	0.58(0.39–0.88,0.01)	0
	Bladder preservation modality	BCG	4	0.71(0.46–1.10,0.13)	0
		Mix	1	0.38(0.16–0.88,0.02)	not available
	Age	< 65 years old	1	1.36(0.35–5.29,0.65)	not available
		> 65 years old	4	0.58(0.39–0.87,0.009)	0
10-yr	All included studies		4	0.62(0.43–0.88,0.007)	0
	Age	< 65 years old	1	1.13(0.34–3.79,0.84)	not available
		> 65 years old	3	0.58(0.40–0.84,0.004)	0
15-yr	All included studies		1	0.35 (0.13–0.95,0.04)	not available

### Comparison of cancer-specific survival of bladder preservation versus radical cystectomy

Data for CSS was extractable from 9 studies and the combined HR was 0.91 (95%CI = 0.44–1.87, *P* = 0.79) synthesizing from 6 of them having significant heterogeneity (*I*^2^ = 83%) (Fig. 3b, Table 3). All subgroups presented insignificant differences between the two management strategies and significant heterogeneities expect for patients younger than 65 years old (*P* = 0.05, *I*^2^ = 0%), and receiving RC for more than 3 months from diagnosis was only present in one single study (*P* = 0.02) (Table 3).

**Fig. 5:**
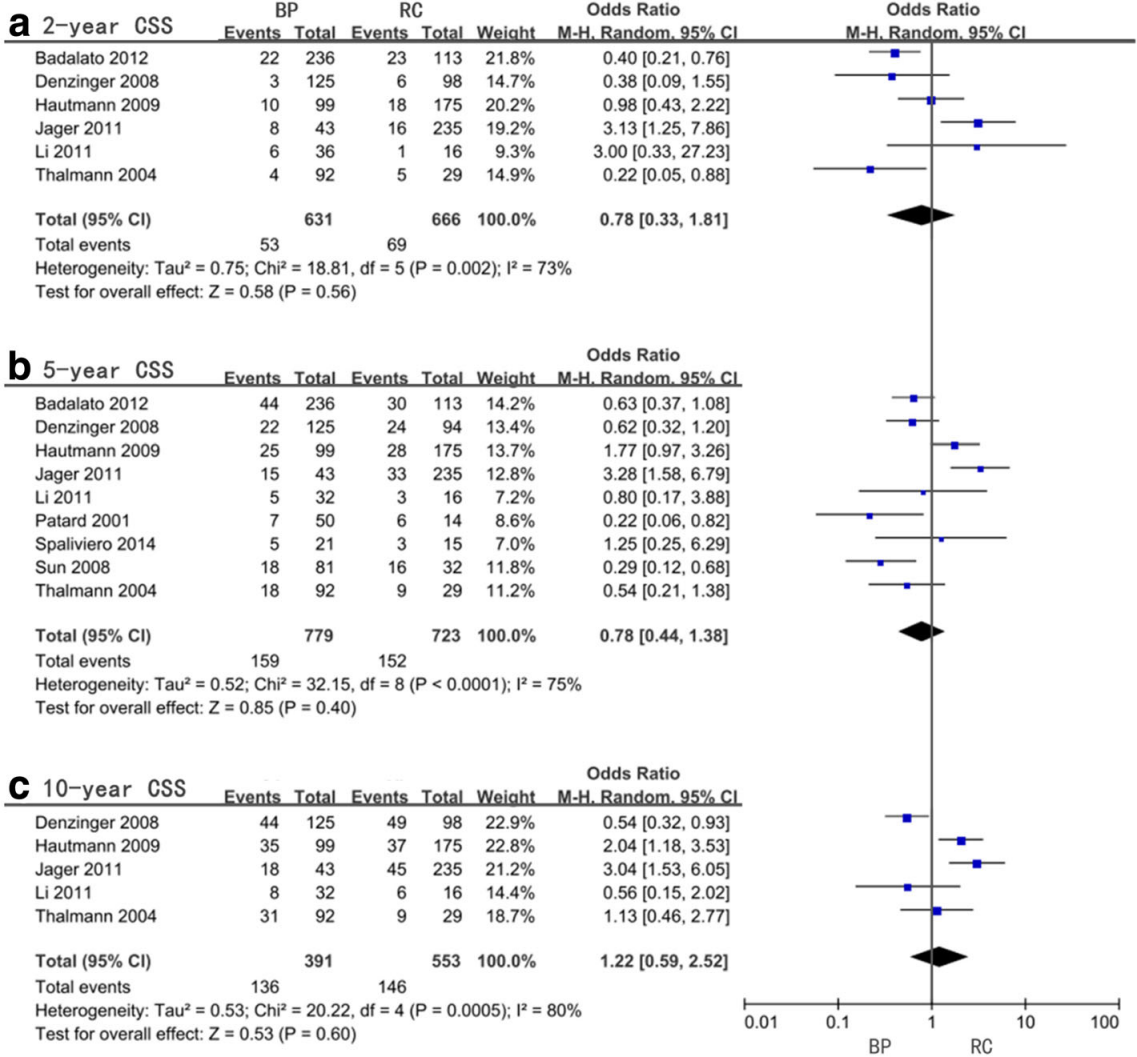
Forest plots of odds ratio (OR) for cancer-specific survival (CSS)

Insignificant differences were observed from the pooled OR and 95%CI for 2-year, 5-year, and 10-year CSS which were 0.78 (0.33–1.81, *P* = 0.56, *I*^2^ = 73%), 0. 78 (0.44–1.38, *P* = 0.4, *I*^2^ = 75%), and 1.22 (0.59–2.52, *P* = 0.6, *I*^2^ = 80%), respectively (Fig. 5, Table 5). The analysis of the 15-year CSS reflected significant differences with an OR = 2.19 (95%CI = 1.21–3.95, *P* = 0.009); however, the heterogeneity was significant (*I*^2^ = 55%), and subgroup analysis was unable to be performed. All subgroup analysis failed to confirm any significant differences for 2-year CSS expect for a subgroup with only one study in favor of RC surgery after more than 3 months from diagnosis versus BP (*P* = 0.02). For the 10-year CSS, a statistical significance suggesting RC performed more than 3 months from diagnosis could reduce BC mortality compared to BP was observed and was the same study as RC timing subgroup analysis in HR and 2-year OS (*P* = 0.002). As for the subgroup analysis of original tumor states and bladder preservation modality in 10-year CSS, HG group (*P* = 0.002) and mix group (*P* = 0.002) shared the same statistical significance preferring RC as a superior treatment. Unlike OS, no significant difference was observed with age (Table 5).

**Table 5:** Meta-analysis of odds ratio (OR) for cancer-specific survival (CSS)

CSS	Factors		Study size	OR (95%CI, *P* value)	*I*^2^ (%)
2-yr	All included studies		6	0.78(0.33–1.81,0.56)	73
	Original tumor state	HG	2	1.09(0.15–8.15,0.93)	92
		G3	4	0.63(0.25–1.60,0.33)	48
	Radical cystectomy timing	> 3 months	1	3.13(1.25–7.86,0.02)	not available
		< 3 months	4	0.59(0.26–1.32,0.2)	56
	Bladder preservation modality	BCG	4	0.63(0.25–1.60,0.33)	48
		Mix	2	1.09(0.15–8.15,0.93)	92
	Age	< 65 years old	2	1.12(0.52–2.41,0.77)	0
		> 65 years old	4	0.60(0.18–1.98,0.4)	82
5-yr	All included studies		9	0.78(0.44–1.38,0.4)	75
	Original tumor state	HG	3	1.37(0.40–4.67,0.62)	84
		G3	6	0.59(0.30–1.16,0.13)	70
	Radical cystectomy timing	> 3 months	2	0.89(.06–2.87,0.93)	92
		< 3 months	5	0.91(0.52–1.58,0.73)	49
	Bladder preservation modality	BCG	5	0.70(0.35–1.41,0.32)	65
		Mix	4	0.91(0.31–2.71,0.87)	85
	Age	< 65 years old	3	0.74(0.19–2.83,0.66)	76
		> 65 years old	6	0.78(0.39–1.55,0.47)	77
10-yr	All included studies		5	1.22(0.59–2.52,0.6)	80
	Original tumor state	HG	1	3.04(1.53–6.05,0.002)	not available
		G3	4	0.96(0.45–2.03,0.91)	76
	Radical cystectomy timing	> 3 months	1	3.04(1.53–6.05,0.002)	not available
		< 3 months	3	1.51(0.97–2.34,0.07)	48
	Bladder preservation modality	BCG	4	0.96(0.45–2.03,0.91)	76
		Mix	1	3.04(1.53–6.05,0.002)	not available
	Age	< 65 years old	2	1.22(0.35–4.24,0.75)	70
		> 65 years old	3	1.22(0.41–3.65,0.73)	87
15-yr	All included studies		3	2.19(1.21–3.95,0.009)	55

### Comparison of progression-free survival of bladder preservation versus radical cystectomy

Four studies provided data for PFS and three of them had data of HR. No statistical significance was reported between the two treatment modalities (HR = 0.71, 95%CI = 0.38–1.34, *P* = 0.3) with insignificant heterogeneity (*I*^2^ = 0%) (Fig. 3c, Table 3). The subgroup analysis was conducted for radical cystectomy timing and age but no significant difference was found (Table 3).

**Fig. 6:**
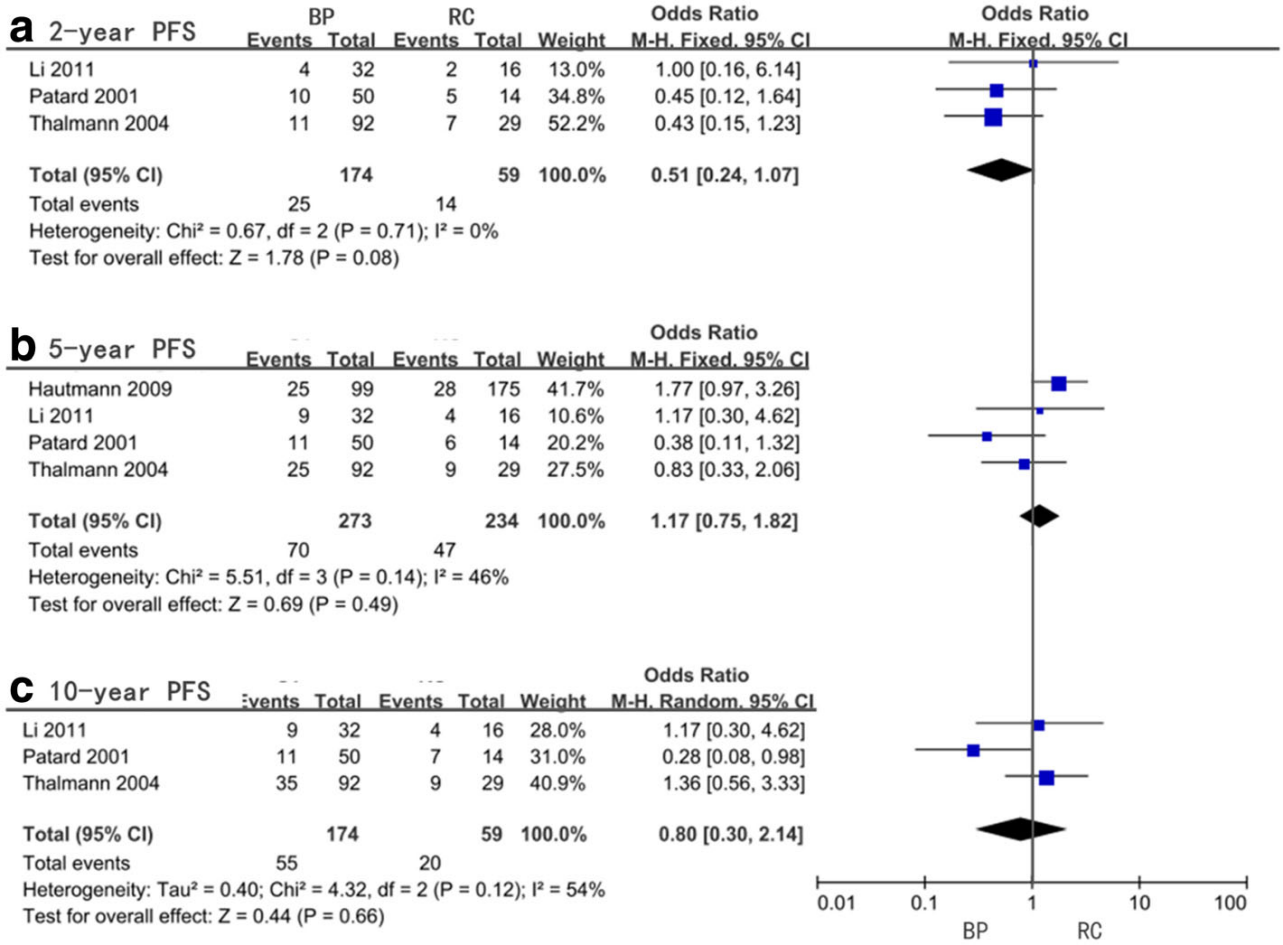
Forest plots of odds ratio (OR) for progression-free survival (PFS)

For the 2-year, 5-year, 10-year, and 15-year PFS, our meta-analysis showed no statistical significance and the pooled OR and 95%CI were 0.51 (0.24–1.07, *P* = 0.08), 1.17 (0.75–1.82, *P* = 0.49), 0.80 (0.30–2.14, *P* = 0.66), and 1.07 (0.08–13.68, *P* = 0.96), respectively (Fig. 6, Table 6). With respect to 2-year PFS (*I*^2^ = 0%) and 5-year PFS (*I*^2^ = 46%), no significant heterogeneity was found while the 10-year PFS (*I*^2^ = 54%) and 15-year PFS (*I*^2^ = 91%) were significant. Limited subgroup analysis was performed and none of them showed any significant differences (Table 6).

**Table 6:** Meta-analysis of odds ratio (OR) for progression-free survival (PFS)

PFS	Factors		Study size	OR (95%CI, *P* value)	*I*^2^ (%)
2-yr	All included studies		3	0.51(0.24–1.07,0.08)	0
	Radical cystectomy timing	> 3 months	1	0.45(0.12–1.64,0.23)	not available
		< 3 months	2	0.54(0.22–1.34,0.18)	0
5-yr	All included studies		4	1.17(0.75–1.82,0.49)	46
	Radical cystectomy timing	> 3 months	1	0.38(0.11–1.32,0.13)	not available
		< 3 months	3	1.37(0.85–2.21,0.2)	0
	Age	< 65 years old	3	1.03(0.40–2.63,0.95)	58
		> 65 years old	1	0.83(0.33–2.06,0.69)	not available
10-yr	All included studies		3	0.80(0.30–2.14,0.66)	54
	Radical cystectomy timing	> 3 months	1	0.28(0.08–0.98,0.05)	not available
		< 3 months	2	1.31(0.62–2.76,0.48)	0
	Age	< 65 years old	2	0.56(0.14–2.26,0.41)	56
		> 65 years old	1	1.36(0.56–3.33,0.49)	not available
15-yr	All included studies		2	1.07(0.08–13.68,0.96)	91

### Publication bias

**Fig. 7:**
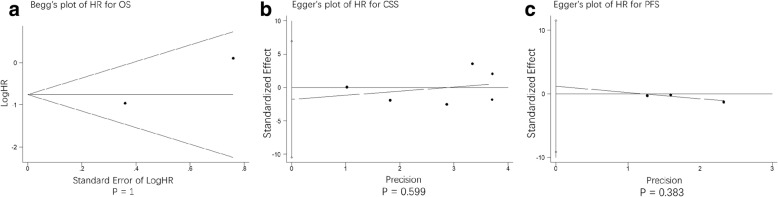
publication bias assessment of hazard ratio (HR)

**Fig. 8:**
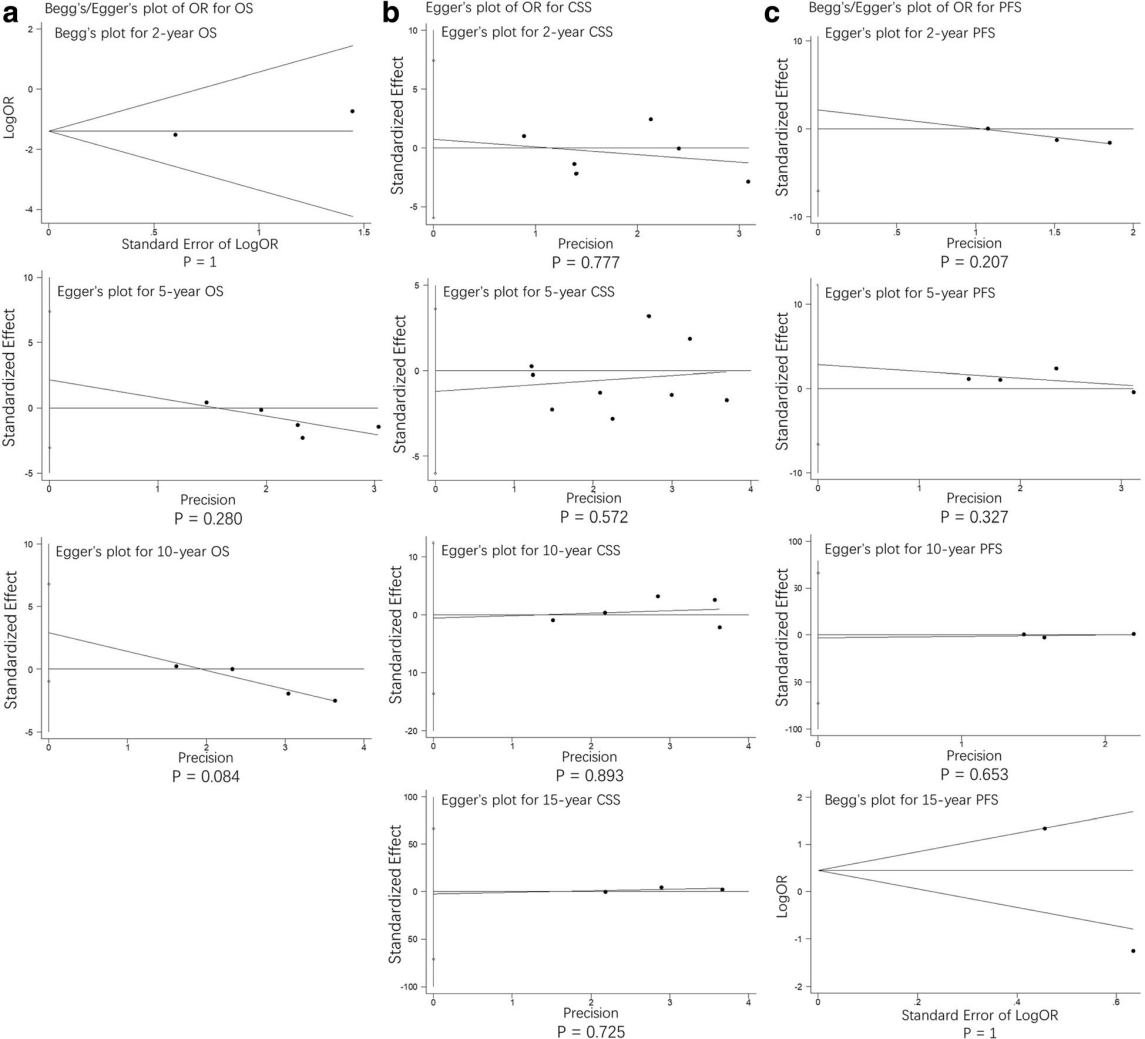
publication bias assessment of odds ratio (OR)

No evidence for publication bias was present for OS (*P* = 1), CSS (*P* = 0.599), and PFS (*P* = 0.383) (Fig. 7) as well as all the specific year groups. (Fig. 8).

## Discussion

Intravesical BCG was first used as a treatment for superficial bladder cancer in 1976 [47]. Since then, this treatment has gradually become the gold standard and stood the test of time for decades. Nowadays, several new bladder preservation approaches have emerged to replace BCG by providing comparable therapeutic efficacy and better quality of life. However, with reference to HGNMIBC regarding high risk of recurrence, progression, and disease-specific death, radical cystectomy still cannot be ruled out during the treatment selection procedure.

In order to provide convincing therapeutic evidence, we performed this meta-analysis to compare BP and RC in HGNMIBC patients. HR was used to compare treatment modality risk through time. The 2-year, 5-year, 10-year, and 15-year OR were used to assess the short-term to long-term survival outcome.

Firstly, all OR and HR of OS reflected significant difference in favor of BP as recommend in AUA, EAU, and NCCN guidelines. As for the subgroup analysis, approximately all groups showed post-BP patients having better survival compared to post-RC patients except for RC patients younger than 65 years old in 5-year and 10-year OS where overall survival advantage versus BP was not statistically significant. However, only several subgroup analyses showed significant differences supporting BP including the G3 group (5-year OS) and > 65-year-old group (5-year and 10-year OS). Hence, we concluded that BP was a more effective treatment modality for HGNMIBC especially for older patients and patients with G3 state tumors. The recommendation has been made that BC patients with failure of local therapy or progression should receive BP following delayed RC rather than immediate RC [48]. The superiority of mixed BP modality compared to RC for 5-year OS could indicate the inferiority of BCG, yet the strength of the evidence is very low. Regarding the original tumor state, the difference in mid-term overall survival may be affected only in T1 patients in the G3 group.

Unlike the OS analysis, the results of CSS failed to confirm any significant difference except for the 15-year CSS, which suggested RC was a better management option for long-term results. The different results between OS and CSS could be explained due to RC causing non-BC mortality. We observed that OR of CSS gradually increases over time. This result may indicate that the complication of surgery has a great impact and decreases over time. A previous long-term study reported 28% of early complications for RC and caused 2.5% of peri-operative deaths [35]. Including from what we observed from the OS results, RC may be a better treatment option for younger patients with better life expectancy, while BP is a better option for elderly patients [49]. The subgroup analysis of radical cystectomy timing showed that RC surgery performed after more than 3 months from diagnosis was a better treatment opinion compared to BP in the subgroup analysis of HR and OR (2-year and 10-year CSS) in the same single study. Therefore, these results may further prove the advantage of BP following a delayed RC. BCG was considered a better option compared to mix of BP modality, i.e., compared to RC separately in the 10-year CSS, which was opposite to the result of the 5-year OS; however, both had a low degree of evidence strength. But this paradoxical result could be explained by the non-cancer-specific death in RC as well. Although a meta-analysis reported that no statistical significance was found in survival outcome between BCG and MMC [50], recent randomized controlled studies have confirmed that BCG has a better recurrence prevention compared to epirubicin [51], epirubicin+interferon [52], or MMC [53]. Hence, BP modality selection is still complex and unclear. Hence, we may need to weigh the advantages and disadvantages of different BP before selection. Regarding original tumor state, RC showed a statistically significant better result for the 10-year CSS in the HG group. This could be explained by CIS, as a confirmed risk factor that decreases survival rates in high-grade tumors [54]. Hence, a more aggressive treatment modality like RC may be more suitable but has to be confirmed in more studies.

Intravesical BCG and RC are considered progression-reducing modalities in separate studies for BC [55, 56]. When comparing the two treatment modalities, the HR and OS results in PFS showed no significant difference with all subgroup analysis having limited quantities due to lack of data. Hence, both BP and RC may have the same effect on preventing disease progression.

In addition to survival outcome, quality of life was mostly affected by the side-effects and complications of treatment and is a crucial determinant for deciding a management modality. Body image and emotional and psychosocial stress are well-known psychosocial side-effects of RC, and more than 80% of patients receiving RC complained of sexual dysfunction [57, 58]. More importantly, severe complications such as fistulas often require surgical repair and sometimes could result in death [59, 60]. As for BP, the side-effect of BCG are usually mild; however, less than 5% of patients can present with severe systemic complications and may even be life-threatening [61]. When choosing the optimal management for HGNMIBC, both survival outcome and QoL outcome need to be weighted according to patients’ characteristics and life expectancy. In addition, the necessity of close follow-up is unarguable and recommended by AUA, EUA, and NCCN guidelines. Even for RC patients, strict long-term monitoring and continuous follow-up are indispensable for monitoring high-risk recurrence and progression [13]. Early detection following early treatment will lead to better survival outcome.

Several significant heterogeneities were observed which could be explained by the differences among studies such as study size, timing of radical cystectomy, bladder preservation modality, and follow-up time. Generally, a larger sample size or longer follow-up time represented a more stable outcome. Among the included studies, heterogeneity could be a result of radical cystectomy performed within or more than 3 months after diagnosis and bladder preservation modality, which varied from study to study. Characteristics of tumor (unifocal or multifocal, size, and grade) and patients (race, age, gender, and risk classification) could also contribute to the heterogeneity results. Multifocal, larger size, and higher grade result in a greater likelihood of a malignant behavior. Differences in race, age, gender, and risk classification may also cause a difference in disease feature and therapeutic responsiveness. Older patients and high-risk tumors usually have the worse prognosis. In addition, the difference in skill level of physician and pathologist will strongly affect the diagnosis and treatment and needs to be considered as an influencing factor.

There were several limitations to our analysis. First, no randomized control trials were found during our searching process probably because of ethical reasons; hence, only included retrospective cohort studies were used for our analysis. Second, differences among eligible studies were inevitable. We selected studies conducted worldwide; hence, the race of the patients were variable. Multiple therapeutic agents and management schedules were used in the BP group. In addition, the observation was included which may cause the BP effect to vary from study to study. Additionally, the use of either the 1973 WHO grading system or 2004 revision may have affected the results of our meta-analysis. Third, as outlined by the NOS scale, some of the included studies failed to control additional factors for comparability, had a short-term follow-up, or did not describe the follow-up schedule and hence contributed to the bias of the studies. Fourth, due to missing or lack of data, some analyses were performed on limited studies, and several subgroup analysis were unable to be performed. Fifth, disease recurrence is a very important outcome but no study provided such data for analysis.

## Conclusion

From our meta-analysis, we concluded that although having the same effect of preventing cancer-specific death and progression from HGNMIBC, bladder preservation approach is a superior modality compared to radical cystectomy. It provides better overall survival outcome, especially for the elderly and patients with T1G3 tumor. However, choosing an optimal BP strategy is still unclear and indefinite. For patients with expected longer life expectancy, RC could be a better option (more specifically, RC performed more than 3 months from diagnosis). However, this conclusion is from very limited data, which needs to be verified using future studies. During the decision-making process of selecting a treatment modality, quality of life should be considered equal to survival outcome. Last but not the least, a close follow-up is essential for any treatment modality.

Heterogeneity and limitations were inevitable and decreased the reliability of our study. Hence, a high quality, prospective, randomized study comparing the effectiveness and the tolerability of BP versus RC is extremely necessary in the future. There are protocols for randomized controlled feasibility studies designed to compare intravesical BCG and RC for high-risk non-muscle-invasive bladder cancer [62]. With the development of multiple BP modalities and reducing surgery, associated death rate will help develop an excellent curative effect and quality of life.

## Abbreviations

AJCC: American Joint Committee on Cancer
AUA: American Urological Association
BC: Bladder cancer
BCG: Bacillus Calmette–Guerin
BP: Bladder preservation
CI: Confidence interval
CIS: Carcinoma in situ
CNKI: China National Knowledge Infrastructure
CSS: Cancer-specific survival
cT1: Clinical T1
cTa: Clinical Ta
CTC: Circulating tumor cells
EAU: European Association of Urology
EPI: Epirubicin
G1: Grade 1
G2: Grade 2
G3: Grade 3
HCPT: Hydroxycamptothecine
HG: High-grade
HGNMIBC: High-grade non-muscle-invasive bladder cancer
HR: Hazard ratio
IVT: Intravesical therapy
LG: Low-grade
MMC: Mitomycin
mo: Month
NCCN: National Comprehensive Cancer Network
NMIBC: Non-muscle-invasive bladder cancer
NOS: Newcastle-Ottawa Scale
OR: Odds ratio
OS: Overall survival
PFS: Progression-free survival
QoL: Quality of life
RC: Radical cystectomy
T1: Tumor invades lamina propria
Ta: Noninvasive papillary carcinoma
TNM: Tumor node metastases
TUR: Transurethral resection
un: Unknown
WHO: World Health Organization
yr: Year
